# Chromosome-scale assembly and quantitative trait locus mapping for major economic traits of the *Culter alburnus* genome using Illumina and PacBio sequencing with Hi-C mapping information

**DOI:** 10.3389/fgene.2023.1072506

**Published:** 2023-05-25

**Authors:** Shili Liu, Jianbo Zheng, Fei Li, Meili Chi, Shun Cheng, Wenping Jiang, Yinuo Liu, Zhimin Gu, Jinliang Zhao

**Affiliations:** ^1^ Key Laboratory of Freshwater Aquaculture Genetic and Breeding of Zhejiang Province, Zhejiang Institute of Freshwater Fisheries, Huzhou, China; ^2^ Key Laboratory of Freshwater Aquatic Genetic Resources, Ministry of Agriculture, Shanghai Ocean University, Shanghai, China; ^3^ Shanghai Engineering Research Center of Aquaculture, Shanghai Ocean University, Shanghai, China; ^4^ National Demonstration Center for Experimental Fisheries Science Education, Shanghai Ocean University, Shanghai, China

**Keywords:** Culter alburnus, genomes, *de novo* sequencing, quantitative trait loci, single nucleotide polymorphisms

## Abstract

Topmouth culter (*Culter alburnus*) is an economically important freshwater fish with high nutritional value. However, its potential genetic advantages have not been fully exploited. Therefore, we aimed to determine the genome sequence of *C. alburnus* and examine quantitative trait loci (QTLs) related to major economic traits. The results showed that 24 pseudochromosomes were anchored by 914.74 Mb of the *C. alburnus* genome sequence. *De novo* sequencing identified 31,279 protein-coding genes with an average length of 8507 bp and average coding sequ ence of 1115 bp. In addition, a high-density genetic linkage map consisting of 24 linkage groups was constructed based on 353,532 high-quality single nucleotide polymorphisms and 4,710 bin markers. A total of 28 QTLs corresponding to 11 genes, 26 QTLs corresponding to 11 genes, and 12 QTLs corresponding to 5 genes were identified for sex, intermuscular spine number and body weight traits, respectively. In this study, we assembled an accurate and nearly complete genome of *C. alburnus* by combining Illumina, PacBio, and high-throughput Chromosome conformation capture (Hi-C) technologies. In addition, we identified QTLs that explained variances in intermuscular spine number, body weight, and sex differences in *C. alburnus*. These genetic markers or candidate genes associated with growth traits provide a basis for marker-assisted selection in *C. alburnus.*

## 1 Introduction

The topmouth culter (*Culter alburnus*, 2n = 2× = 48), is a member of Cyprinidae, Cultrinae, *Culter*, and has high economic and nutritional values (Ren et al., 2014). Topmouth culter are widely distributed in the lakes and reservoirs of China (Liu et al., 2019). In the past 10 years, with the breakthroughs in artificial breeding and breeding technology, *C. alburnus* has been cultivated on a large scale in more than ten provinces and cities, with an output value of nearly 10 billion yuan (Wang et al., 2007). In addition, *C. alburnus* is at the top of the food chain in the freshwater ecosystem, playing important roles in maintaining the stability of the ecosystem. However, most of the species used for breeding are wild species that have not been bred, and their potential genetic advantages have not been fully exploited (Li et al., 2010). In addition, blind breeding, introduction, hybridization, and repeated breeding have caused the growth of the cultivated *C. alburnus* to slow down, resulting in individual germplasm decline or confounding phenomena, such as miniaturization. Therefore, germplasm improvement has become an urgent need for the sustainable and healthy development of the topmouth culter breeding industry.

To date, research on *C. alburnus* has mainly focused on nutrition, growth, artificial reproduction, pond culture, and genetic diversity (Liu et al., 2014; Xiao et al., 2014; Sun et al., 2015). From the perspective of breeding, although *C. alburnus* has not shown an obvious economic decline phenomenon, molecular studies on the genetic diversity of breeding populations have shown that the genetic diversity of *C. alburnus* is at a low level (Qi et al., 2013; Qi et al., 2015). To prevent the decay of the germplasm resources of *C. alburnus* and further tap the potential of its breeding traits, breeding work on *C. alburnus* is particularly important.

Meat quality is affected by many factors, including environmental, nutritional, and genetic factors (Pathare and Roskilly, 2016). Research on marine meat quality has lagged behind that of livestock and poultry. In recent years, the analysis of fish meat quality has been limited to the analysis of conventional nutrients, amino acids, and fatty acids (Wang et al., 2006; Calanche et al., 2019). With the rapid development of molecular biology technology, the mechanisms determining meat quality can be further studied at the molecular level, and the problem of meat quality improvement can be solved.

Growth is one of the most critical economic traits for fish (Feng et al., 2018). Multiple gene quantitative trait loci (QTLs) involving environmental and genome effects determine the growth trait (Feng et al., 2018). Genetic maps, the inheritance patterns of traits, and genetic markers have been developed and used for a wide range of species, including fish (Rexroad et al., 2008). These tools target the discovery of allelic variation that affects traits, with an ultimate goal of identifying DNA sequences underlying phenotypes (Rexroad et al., 2008). QTL mapping has identified many important traits for sex and growth in fish (Mackay et al., 2009). Traditional selective breeding methods have encountered some difficulties, such as uncertainty, extensive workload, being time-consuming, and slow to take effect. Hence, molecular breeding methods are needed to accelerate the breeding process in fish.

In this study, we applied a combined strategy involving Illumina, PacBio, and high-throughput Chromosome Conformation Capture (Hi-C) technologies to generate sequencing data for the chromosomal genome construction of *C. alburnus*. We identified putative genes associated with sex, intermuscular spine number, and body weight. In addition, we identified the QTLs explaining variances in intermuscular spine number, body weight, and sex differences in the *C. alburnus*. The results of this study will provide support for the molecular breeding of *C. alburnus*.

## 2 Materials and methods

### 2.1 Sample collection, library construction, and sequencing

A healthy female *C. alburnus* collected from the Balidian experimental base of Zhejiang Institute of Freshwater Fisheries was used for whole-genome sequencing. Total genomic DNA was extracted from a tail fin sample using the cetyltrimethylammonium bromide (CTAB) method. A whole-genome shotgun sequencing strategy was applied, and short-insert libraries (250, 500, and 800 bp) and long-insert libraries (2 and 5 kb) were constructed using the standard protocols provided by Illumina (San Diego, CA, United States). The resulting libraries were sequenced using the Illumina HiSeq X Ten platform. Furthermore, two 10 kb libraries were also constructed from the extracted DNA samples following the PacBio manufacturing protocols (Pacific Biosciences, Menlo Park, CA, United States). Subsequently, the libraries were sequenced with two cells based on the PacBio Sequel platform. Meanwhile, the same sample was also used to construct a Hi-C sequencing library. The Hi-C procedure was performed as described in previous studies (Zhang et al., 2019; Shan et al., 2020). Briefly, the extracted DNA was randomly broken into 300–500 bp fragments and labeled with biotin. The biotin-labeled fragments were extracted using magnetic beads. The Hi-C library was finally built after end repair, adding a poly-A tail, adapter ligation, PCR amplification cycle evaluation, and library purification. The Hi-C library was preliminarily quantified using a Qubit2.0 fluorometer (Themo Fisher Scientific, Waltham, MA, United States) and the insert size was determined using Agilent 2,100 instrument (Agilent Technologies, Santa Clara, CA, United States). The qualified library was sequenced on the Illumina Hiseq X ten platform to produce 150 bp paired-end reads.

The QTL analysis was performed on the male and female parental generation (F_0_) and the full-sib F_1_ offspring, including 101 females and 99 males. The figerlings of *C. alburnus* were stocked in an earthen pond (35 m × 35 m × 1.5 m) with a stocking density of 22500 fish/ha. They were fed with commercial extruded feed (Minghui Feed Co. LTD) containing 41% crude protein. No other special treatments were applied. Growth-related traits including body weight (g), body length (cm), body width (cm), head length (cm), total length (cm), and the number of intermuscular spines were collected after 580 days of feeding trials. The distribution patterns of these traits were calculated using the Kolmogorov–Smirnov test in GraphPad Prism (version 5.0; GraphPad Inc., La Jolla, CA, United States). These growth-related traits exhibited high variation in this family and therefore could be used for linkage construction and QTL analysis. Genomic DNA of the male and female parents and the 200 progeny was extracted and Illumina sequencing using the HiSeq 4,000 platform was performed by Origingene company (Shanghai, China).

### 2.2 Genome size estimation and *de novo* genome assembly

The genome size of *C. alburnus* was estimated based on the routine 17-mer depth frequency distribution analysis (Liu et al., 2013) using the clean reads from the short-insert libraries. Subsequently, a *de novo* genome assembly was generated using both the Illumina short reads and the PacBio long reads. First, the Illumina sequencing data were assembled into contigs using default parameters by Platanus version 1.2.4 (Kajitani et al., 2014). Next, the Platanus-generated contigs, together with the PacBio reads, were used to generate a hybrid assembly by DBG2OLC with default parameters (Ye et al., 2016). The obtained scaffolds were then polished using Pilon (Walker et al., 2014) to generate the final genome assembly of *C. alburnus*. Finally, the completeness of the genome sequence was evaluated using benchmarking universal single-copy orthologs (BUSCO) version 3.0 (Simão et al., 2015) together with the Actinopterygii_odb9 database.

### 2.3 Chromosome-level assembly of the Hi-C data

The raw Hi-C sequence data was subjected to quality control to obtain clean data, including removing adapters, the non-AGCT bases at the 5′ end, and low-quality reads. The clean reads were aligned against the genome assembled by PacBio sequencing using BWA (v 0.7.13) software with default parameters (Li and Durbin, 2010). For the Hi-C assembly, data were first processed using HiC-Pro v2.8.023 (default settings) (Servant et al., 2015), and then Juicer v1.524 (default settings) (Durand et al., 2016) was used to map the reads onto the genome to validate the available paired reads. The 3D-DNA pipeline v18092225 (parameters: m haploid -r 2) (Dudchenko et al., 2017) was used to construct a candidate chromosome-level genome. Finally, we reviewed the candidate assembly using juicebox assembly tools (Dudchenko et al., 2018) for quality control and for interactive correction of the automatic output, ultimately obtaining the final assembly.

### 2.4 Genome-wide evolutionary analysis and functional annotation

The coding gene sequences of 12 species of fish (*Anabarilius grahami*, *Ctenopharyngodon idellus*, *Cyprinus carpio*, *Danio rerio*, *Gadus morhua*, *Gasterosteus aculeatus*, *Oryzias latipes*, *Petromyzon marinus*, *Takifugu rubripes* and *Megalobrama amblycephala*) were downloaded from Ensembl or GenBank databases. Then, based on the OrthoMCL process (Li et al., 2003), a homologous gene search was performed, and the gene sequence supermatrix was constructed using RAxML software with 1,000 rapid bootstrap analyses to build a maximum-likelihood (ML) phylogenetic tree (Stamatakis, 2014). In addition, the divergence time of the 13 species was estimated using MCMCtree in the PAML package (Yang, 1997). Expansion and contraction of genes were analyzed using CAFE (version 2.1) (https://github.com/hahnlab/CAFE) calculation based on changes in gene cluster size and the species divergence time.

Gene annotation was performed by combing results of *de novo* analysis, homolog analysis, and RNA-seq assisted prediction. The homologous genes of five species, including *C. alburnus*, *Cyprinus carpio*, *Danio rerio*, *Gadus morhua*, and *Megalobrama amblycephala* were compared. The sequences of protein-coding genes were searched against the NCBI non-redundant Protein database (NR), String Protein database, Swiss-Prot protein database as well as Protein Families (PFAM), Kyoto Encyclopedia of Genes and Genomes pathway database (KEGG), and Gene Ontology (GO) using BlastX (Verison 2.2.25, E value < 1e^−5^). The GO annotations were determined using Blast2GO to obtain the functional classification of the unigenes (Conesa et al., 2005).

### 2.5 Single nucleotide polymorphism (SNP) calling

The raw reads were quality controlled to remove adaptors and low-quality reads to obtain clean reads. The high-quality clean reads were pre-processed, including mark duplicates, local realignment, and base recalibration, according to their location in the reference genome. Then, the SNPs was determined using the GATK software tool according to the instructions at URL (https://software.broadinstitute.org/gatk/best-practices/).

The SNPs of all samples were combined. Only the homozygous and different loci of the parents were retained, and the SNP loci with an uncertain genotype in the parents were filtered out. In addition, the low-quality SNPs including: 1) Quality < 30.0, logarithm of odds (LOD) threshold < 1.0, Fisher strand (FS) > 60.0, map quality (MQ) < 2.0, Strand Odds Ratio (SOR) > 8.0 and ReadPosRankSum< −8.0; 2) over-dense SNPs; 3) SNPs covering less than 50% of all offspring; and 4) SNPs with a minimum allele frequency (MAF) < 0.05 were filtered out. Finally, the high-quality SNP tags were obtained to construct genetic linkage maps.

### 2.6 Construction of linkage groups and QTL analysis

Linkage groups were constructed by calculating the recombination rate between pairs of tags. Linkage map construction was performed using JoinMap version 4.0 (Stam, 1993). Three traits, including body weight, sex, and intermuscular spine number were analyzed. The QTL analysis was conducted employing ICIM mapping 4.1 software (Meng et al., 2015). The significance criteria were set according to the permutation test (1,000 replicates). The confidence interval (CI) was first set at 99% to determine the LOD threshold. If there was no QTL at this threshold, the 95% CI and 90% CI would be considered. If there was still no QTL even at 90% CI, the LOD threshold was set as 3.0, then reduced to 2.5 and then to 2.0 to finally obtain the QTLs.

## 3 Results

### 3.1 Assembly of the *C. alburnus* genome using illumina and PacBio sequencing

We generated ∼192.54 Gb [298.31 Gb of Illumina plus 34.36 Gb of Pacific Biosciences (PacBio) reads] of sequencing data for *C. alburnus* (Supplementary Tables S1, S2). The resulting assembly comprised 1,055 Mb, corresponding to 4,122 reads (Table 1). These sizes were very similar to the genome sizes estimated using K-mer analysis. The peak around the depth of 44 represent the homozygous K-mer value. The genome size is 950 Mb according to formula: Genome Size = K-mer_num/Peak_depth (Supplementary Figure S1).

**TABLE 1:** The summary of *C. alburnus* genome.

Scaffold		
max_length: 7138733 bp	Length (bp)	Number
N90	102269	1419
N50	1172915	249
Total length: 1055142460 bp		
Length ≥ 1000 bp	4120	
Contig		
max_length: 7138733 bp	Length (bp)	Number
N90	100972	1454
N50	1152803	256
Total length: 1055115805 bp		
Length ≥ 1000 bp	4230	

The lengths of contig N50 and scaffold N50 were 1.15 Mb and 1.17 Mb, respectively (Table 1). Assessments of the genome assembly quality showed that the assembly covered 92.1% unigenes (4,219) derived from the whole-genome gene datasets of Actinopterygii and had few (5.1%, 238) missing benchmarking sets of universal single-copy orthologs (BUSCOs) (Supplementary Table S3). The final genome assembly and raw data from the PacBio and Hi-C libraries, as well as the annotation, have been deposited at NCBI under BioProject PRJNA700093.

### 3.2 The Hi-C assisted-assembly of the *C. alburnus* genomes

A total of 896,981,652 reads were obtained containing 134,547,247,800 bases. The Q20% and Q30% were 96.3% and 92.3%, respectively and the GC% was 37.7%, indicating relatively high quality sequencing. In total, 366,841,534 (40.9%) reads were mapped to the genome and 86,119,552 (9.6%) of them were effective data.

**FIGURE 1:**
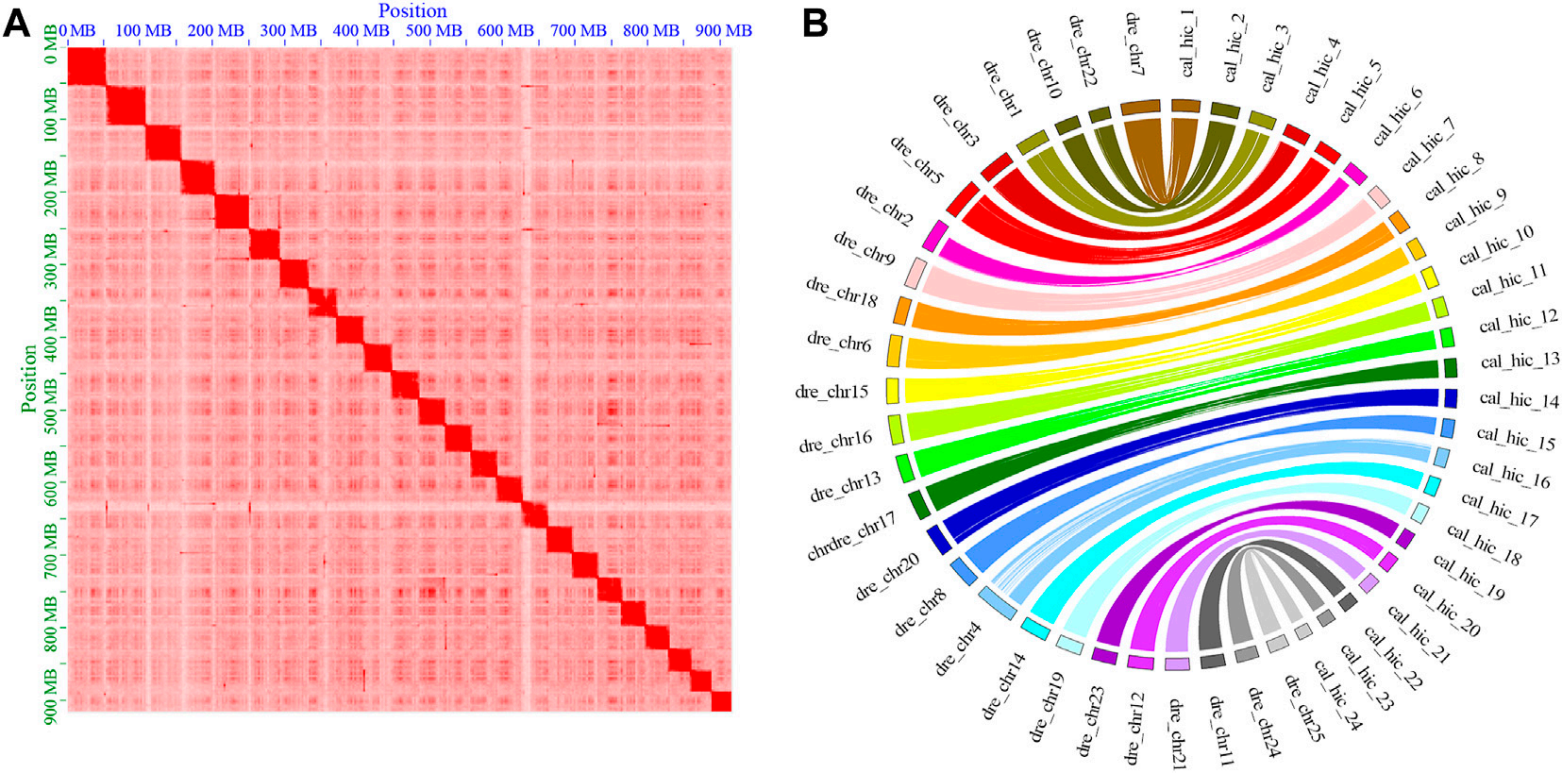
Overview of the assembly quality and characteristics of the *C. alburnus* genome. **(A)** Hi-C interactions among 24 chromosomes with a resolution of 2.5 Mb. The intensity of the interactions increased along with the color from light to dark. The abscissa and ordinate indicate the N*bin position on the genome. The 24 squares in the figure are the 24 pairs of chromosomes of *C. alburnus.* **(B)** Syntenic relationship between *C. alburnus* and *Danio rerio*. Maps of the 24 *C. alburnus* chromosomes and the 25 *Danio rerio* chromosomes based on the positions of highly conserved Syntenic orthologous gene pairs.

In total, 914.74 Mb (86.69%) of the *C. alburnus* sequences with an N50 length of 35.18 Mb were anchored to the 24 pseudo-chromosomes by mapping Hi-C contact data using 2438 contigs (Table 2). A heatmap of chromosome interactions was constructed to visualize the contact intensity among chromosomes (Figure 1A). The number of the pseudo-chromosomes was the same as that of chromosomes, and corresponded to the top 24 longest scaffolds. Then, a genetic interaction map between *C. alburnus* and zebrafish was constructed. Multiple genome alignments showed that the genome of *C. alburnus* had a high degree of collinearity with that of zebrafish (Figure 1B).

**TABLE 2:** Statistics the Hi-C assembly results.

Pseudochromosomes of *Culter alburnus*	Number of contigs	Length of contigs (bp)	Length of pseudochromosomes (bp)	The best-match results in zebrafish chromosomes
Cal_HiC_1	128	53619570	53681983	Dre_chr7
Cal_HiC_2	164	53329318	53408973	Dre_chr10,Dre_chr22
Cal_HiC_3	197	48735587	48831806	Dre_chr1
Cal_HiC_4	211	47261930	47366043	Dre_chr3
Cal_HiC_5	109	46495101	46549101	Dre_chr5
Cal_HiC_6	174	42002982	42088092	Dre_chr2
Cal_HiC_7	90	39740707	39784316	Dre_chr9
Cal_HiC_8	90	38810448	38854547	Dre_chr18
Cal_HiC_9	76	38427020	38463651	Dre_chr6
Cal_HiC_10	125	37446877	37507352	Dre_chr15
Cal_HiC_11	85	37373272	37414394	Dre_chr16
Cal_HiC_12	45	36302149	36324009	Dre_chr13
Cal_HiC_13	59	36005317	36032583	Dre_chr17
Cal_HiC_14	79	35141052	35179483	Dre_chr20
Cal_HiC_15	80	34984608	35023306	Dre_chr8
Cal_HiC_16	179	34791782	34879155	Dre_chr4
Cal_HiC_17	82	34802649	34842297	Dre_chr14
Cal_HiC_18	85	34200354	34241869	Dre_chr19
Cal_HiC_19	79	32853770	32893328	Dre_chr23
Cal_HiC_20	60	32773783	32802556	Dre_chr12
Cal_HiC_21	77	32673307	32710816	Dre_chr21
Cal_HiC_22	46	30401594	30423689	Dre_chr11
Cal_HiC_23	43	28017911	28038508	Dre_chr24
Cal_HiC_24	75	27360271	27403217	Dre_chr25
Total	2438	913551359	914745074	\

### 3.3 Genome evolution analysis of *C. alburnus*

**FIGURE 2:**
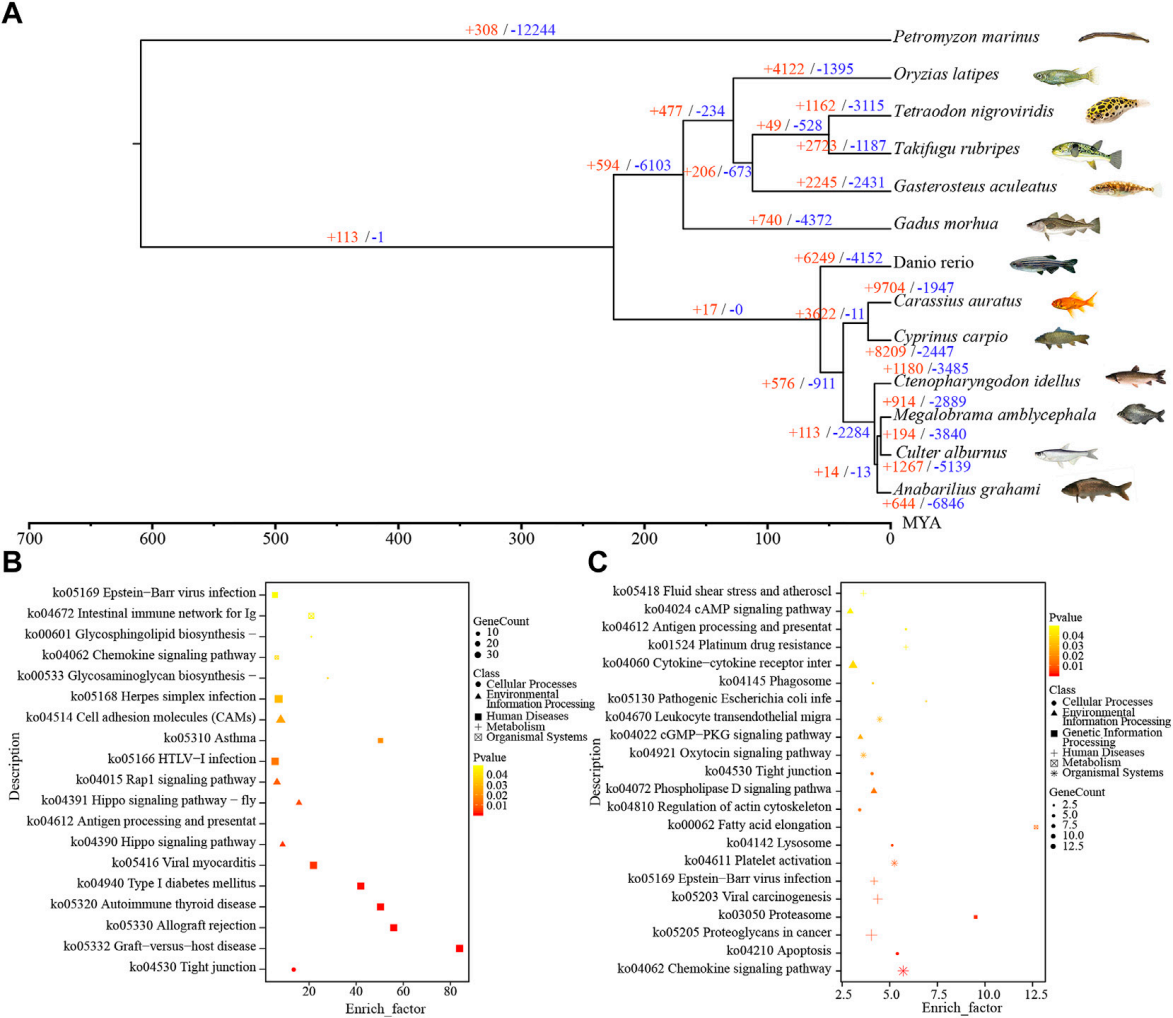
Genome evolution analysis of *C*. a*lburnus*. **(A)** A phylogenetic tree of divergence time, and and contraction events of gene clusters constructed from 13 species. The time of divergence was analyzed using a maximum-likelihood tree. The number of expansion events in each gene cluster is indicated in red, and contraction events are indicated in blue. **(B)** KEGG enrichment analysis of the expansion events of the gene clusters. **(C)** KEGG enrichment analysis of the contraction events of the gene clusters.

To reveal the genome evolution of *C. alburnus* and other related species, we downloaded the coding gene sequences of 12 species of fishes to build a phylogenetic tree. As shown in Figure 2A, the ancestral lineage of *C. alburnus* was closest to *Megalobrama amblycephala* and farthest from *Petromyzon marinus*. *C. alburnus* diverged from *Petromyzon marinus* more than 600 million years ago, and from *M. amblycephala* about 7.7–8.8 million years ago.

Expansion and contraction analysis of gene clusters showed that 1267 expansion events and 5139 contraction events have occurred in *C. alburnus*. Among them, 24 expansion events and 37 contraction events were significant (*p* < 0.05). Then, functional annotation was performed for the expansion and contraction events (Supplementary Tables S4, S5). Kyoto Encyclopedia of Genes and Genomes (KEGG) enrichment analysis indicated that the expansion events were significantly related to “Tight junction” (false discovery rate (FDR = 0.009209), “Hippo signaling pathway” (FDR = 0.047693), and “Antigen processing and presentation” (FDR = 0.047693) (Figure 2B). The contraction events were significantly associated with “Neuroactive ligand-receptor interaction” (FDR = 0.000492), “Sphingolipid signaling pathway” (FDR = 0.003244) and “Chemokine signaling pathway” (FDR = 0.024271) (Figure 2C).

### 3.4 *De novo* genome annotation of *C. alburnus*

**FIGURE 3:**
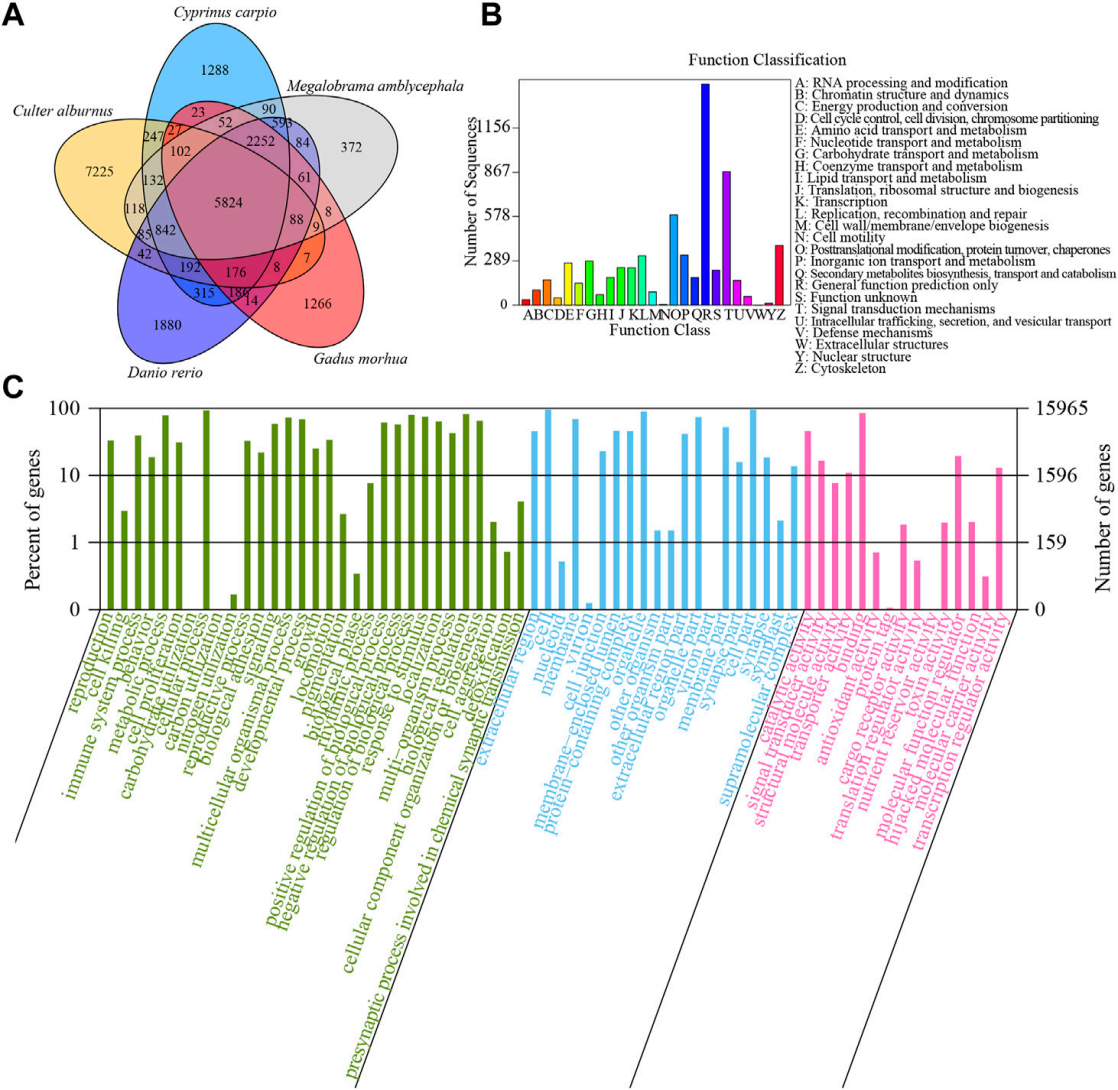
*De novo* genome annotation of *C*. a*lburnus*. **(A)** VENN diagram of homologous genes in *Culter alburnus*, *Cyprinus carpio*, *Danio rerio*, *Gadus morhua* and *Megalobrama amblycephala.* **(B)** COG functional classification statistics. **(C)** GO enrichment analysis.

The homologous genes of five species, including *C. alburnus*, *Cyprinus carpio*, *Danio rerio*, *Gadus morhua*, and *Megalobrama amblycephala* were compared. There were 5824 overlapping homologous genes among these five species (Figure 3A). Based on *de novo*, homolog-based, and RNA-seq approaches, 31,279 protein-coding genes with an average gene length of 8507.7 bp and average coding sequence (CDS) length of 1115.23 bp were identified in the assemblies. The overlapping homologous genes account for 38.51% (5824/15124) of genes in *C. alburnus*.

The mapping rates of protein-coding genes against the GO, KEGG, NR, PFAM, String, and Swissprot databases were 15.30%, 12.80%, 22.80%, 15.50%, 18.00%, and 15.60%, respectively (Supplementary Table S6). Clusters of orthogonal groups (COG) functional classification demonstrated that the most genes were related to “general function prediction,” “Signal transduction mechanisms,” “Posttranslational modification, protein turnover, chaperones” and “Cytoskeleton” (Figure 3B). GO enrichment analysis suggested the genes were related to the biological processes of “reproduction” (GO:0000003), “cell killing” (GO:0001906), “immune system process” (GO:0002376), and “cell proliferation” (GO: 0008283). The proteins encoded by these genes were predicted to be located on “extracellular region” (GO:0005576), “cell” (GO:0005623), “nucleoid” (GO:0009295), and “membrane” (GO:0016020) (Figure 3C). The molecular functions included “catalytic activity” (GO:0003824), “signal transducer activity” (GO:0004871), “structural molecule activity” (GO:0005198), and “transporter activity” (GO:0005215). The top 20 KEGG pathways involving the unigenes included “pathways in cancer” (494 unigenes), “neuroactive ligand-receptor interaction” (431 unigenes), “PI3K-Akt signaling pathway” (415 unigenes), and “Cell adhesion molecules (CAMs)” (400 unigenes) (Supplementary Figure S2).

### 3.5 Non-coding RNA annotation in the genome of *C. alburnus*

Non-coding RNA refers to RNA that does not translate into protein, including rRNA, tRNA, snRNA, and miRNA. These RNAs play important roles in biological regulation. Based on the RNA-seq data, 381 miRNAs, 3622 tRNAs, 1762 rRNAs, and 423 snRNAs were predicted in the *C. alburnus* genome (Table 3).

**TABLE 3:** Non-coding RNA annotation in the genome of *C. alburnus*.

Type		Copy	Average length(bp)	Total length(bp)	% Of genome
miRNA		381	85.93	32738	0.00310
tRNA		3622	76.50	277065	0.02626
rRNA	rRNA	881	169.26	149114	0.01413
	18S	11	2321.73	25539	0.00242
	28S	6	4101.17	24607	0.00233
	8S	864	114.55	98968	0.00938
snRNA	snRNA	212	119.42	25317	0.00240
	CD-box	120	95.92	11510	0.00109
	HACA-box	73	147.60	10775	0.00102
	splicing	9	129.56	1166	0.00011
	scaRNA	9	201.11	1810	0.00017

### 3.6 Characteristics of the phenotypic traits

The mapping family in this study consisted of 200 *C. alburnus* progeny, and the phenotypic growth-related traits all passed the normality test (*p* > 0.05 for all) according to the Kolmogorov–Smirnov test. The average values of bodyweight, total length, body length, body height, and head length were 0.45 ± 0.10 kg, 38.96 ± 2.76 cm, 33.53 ± 2.64 cm, 8.06 ± 0.72 cm, and 7.10 ± 0.47 cm, respectively (Supplementary Table S7). The average total number of intermuscular spines was 132.6 ± 1.954 (Supplementary Table S8). The mapping family included 99 males and 101 females, with a sex ratio of 1:1.02.

### 3.7 QTL *analysis*

After filtering out the low-quality SNPs, 353,532 high-quality SNPs and 4,710 bin markers were retained in the “all” group (Supplementary Figure S3). These bin markers were grouped into 24 linkage groups (LGs). The genetic map spanned 2307.8 cM with an average marker interval of 1.35 cM. The genetic length of the LGs ranged from 47.40 cM (LG12) to 210.80 cM (LG10) with an average of 96.16 cM. The number of markers varied from 38 (LG24) to 177 (LG1) with an average of 81 (Supplementary Figure S3; Supplementary Table S9). For the “female” group, 355,547 high-quality SNPs and 4,240 bin markers were retained. The genetic map spanned 2032.4 cM with an average maker interval of 1.29 cM. The genetic length of the LGs ranged from 32.9 cM (LG24) to 155.6 cM (LG1)with an average of 84.68 cM, and the number of markers varied from 32 (LG24) to 189 (LG1) with an average of 80 (Supplementary Figure S4; Supplementary Table S10). For the “male” group, 354,207 high-quality SNPs and 4,385 bin markers were retained. The genetic map spanned 2514 cM with an average maker interval of 1.17 cM. The genetic length of the LGs ranged from 60.70 cM (LG14) to 200.8 cM (LG2) with an average of 104.58cM, and the number of markers varied from 43 (LG24) to 283 (LG1) with an average of 105 (Supplementary Figure S5; Supplementary Tables S11).

### 3.8 Identification of potential QTL-related genes

The genome-wide LOD significance threshold for the sex trait was 1.5 based on the permutation test. Using ICIM mapping 4.1 software, 28 QTLs associated with sex were detected on 17 LGs (Supplementary Table S12). The LOD scores ranged from 1.5163 to 4.1739 and the phenotypic variance explained (PVE) ranged from 1.0434% to 7.8872%. Eleven genes, including *NLRC3* (encoding protein NLRC3-like), *ZNF836* (encoding zinc finger protein 836-like), *NLRP12* (encoding NACHT, LRR and PYD domains-containing protein 12-like), and *CD163* (encoding scavenger receptor cysteine-rich type 1 protein M130-like), related to sex were identified from these QTL regions. The QTL with the highest PVE corresponded to the gene CUI009080. Annotation indicated that this gene encoded “PREDICTED: uncharacterized protein LOC109087430 (*Cyprinus carpio*)” in the NR database (Supplementary Table S13).

Based on a LOD significance threshold of 1.5 calculated from the permutation test, 26 QTLs associated with intermuscular spine number were detected on 15 LGs (Supplementary Table S13). The LOD scores ranged from 1.5134 to 3.7688 and the PVE ranged from 0.8557% to 3.4099%. Eleven genes, such as *CSNK2A* (encoding casein kinase II subunit alpha-like) and *IAN12* (encoding immune-associated nucleotide-binding protein 12-like) related with intermuscular spine length were identified from these QTL regions. The QTL with the highest PVE (3.4099%) was annotated to a gene encoding “PREDICTED: uncharacterized protein LOC109094915 (*Cyprinus carpio*)” in the NR database.

Based on a significance threshold of 2 calculated from the permutation test, 12 QTLs associated with weight were detected on 10 LGs (Supplementary Table S13). The LOD scores ranged from 2.0402 to 3.3573 and the PVE ranged from 1.9805% to 4.8974%. Five genes, such as *AK9* (encoding adenylate kinase 9) and *TRIM* (encoding tripartite motif-containing protein) related with weight were identified from these QTL regions. The QTL with the highest PVE (4.8974%) was annotated to an intergenic a region. The QTL with the second-highest PVE (3.272%) was annotated to the gene CUI012867, which was predicted to encode “uncharacterized protein LOC107665087” from the NR database.

## 4 Discussion

*C. alburnus* is an important economic fish in China which has been extensively cultured because of its rapid growth and excellent taste. Germplasm improvement has become an urgent need for the sustainable and healthy development of the *C. alburnus* breeding industry *.* In this study, we performed *de novo* sequencing by combining Illumina, PacBio, and Hi-C technologies. Next-generation sequencing is very accurate; however, the read length is short, which has obvious disadvantages for assembling heterozygous and repetitive sequence regions. Third-generation PacBio sequencing can generate long sequences to cross repetitive regions and reduces the impact of heterozygous sites on genome assembly, which can greatly improve the assembly integrity and quality of complex regions. However, the PacBio sequencing data has a high error rate and often needs to be corrected. In this study, we developed five libraries, including 250 bp, 500 bp, 800 bp, 2 kbp and 5 kbp libraries for next-generation sequencing. Illumina sequencing generated 298.31 Gb of data. The PacBio sequencing has 2 cells, and the data output was 34.36 Gb. Previously, Ren et al., assembled the genomes of *C. alburnus* and *Megalobrama amblycephala*. They generated 176.33 Gb, including171.05 Gb of Illumina and 5.28 Gb of PacBio reads (Ren et al., 2019). The nearly complete genome of *C. alburnus* reported in the present study is more accurate than that published by Ren et al.

The phylogenetic tree showed that the ancestral lineage of *C. alburnus* was closest to *Megalobrama amblycephala* and farthest from *Petromyzon marinus*. *C. alburnus* diverged from *Petromyzon marinus* more than 600 million years ago. According to previous studies, the divergence time between *C. alburnus* and *Petromyzon marinus* varied from 455 million years to 695 million years, with a median time of 615 million years (Aris-Brosou and Yang, 2002; Otsuka and Sugaya, 2003; Blair and Hedges, 2005; Roelants et al., 2010; Chen et al., 2012; dos Reis et al., 2015; Gold et al., 2015; Delsuc et al., 2018). We estimated the divergence time between *C. alburnus* and *Megalobrama amblycephala* as about 7.7–8.8 million years, which is within the range of the results reported by previous studies, ranging from 2.3 million years to 12.74 million years (Tao et al., 2010; Wang et al., 2012; Rabosky et al., 2013; Rabosky et al., 2018; Ren et al., 2019). These results suggested our results are reliable and creditable.

In this study, we used the SNPs approach to construct a new genetic linkage map for *C. alburnus.* A total of 4,710 bin markers were grouped into 24 LGs, which was consistent with the chromosome number from the Hi-C results. Currently, whole genome sequences combined with SNP markers are utilized for QTL analysis (Botstein et al., 1980). SNPs are abundant, widely distributed throughout the genome, and are associated with a unique sequence. SNP-based QTL analysis significantly increases the number of markers discovered compared with other methods (Uchino et al., 2018).

There are obvious differences in the growth rate, nutritional value, and other major economic indicators of the males and females of many kinds of fish. The breeding of unisexual groups can reduce the energy consumption of the reproductive process, as well as avoiding the excessive reproduction of fish with a strong reproductive ability, which is beneficial to control breeding density to obtain better economic benefits of breeding. In this study, we characterized the QTLs related with three traits: body weight, sex, and intermuscular spine number. Eleven genes, including *NLRC3*, *ZNF836*, *NLRP12*, and *CD163* related to sex were identified from 28 QTL regions. NLRC3 is a cytoplasmic pattern recognition receptor, which belongs to the Nod-like receptor (NLR) family (Elinav et al., 2011). NLRs play important roles in microbial sensing and lead to the initiation of antimicrobial and antiviral immune responses (Elinav et al., 2011). Cádiz et al. (2020) found three genes previously associated with defense against bacterial pathogens such as *NLRC3*, *PIGR*, and *MAP1S65* in Nile tilapia. *NLRC3* mRNA expression was found to be upregulated in response to challenge with lipopolysaccharide and *Edwardsiella tarda* in Japanese flounder (Li et al., 2016). NLRP12 is an NLRP family member that could regulate nuclear factor kappa B (NF- κB) activation (Lich et al., 2007). These proteins might function in sex-determination. In addition, 11 genes, including *CSNK2A* and *IAN12* were found to be located in intermuscular spine length-determining regions and 5 genes, including *AK9* and *TRIM,* were found to be located in weight-determining regions.

## 5 Conclusion

In conclusion, this study assembled an accurate and nearly complete genome of *C. alburnus* by combing Illumina, PacBio and Hi-C technologies. Twenty-four peudochromosomes were anchored by 914.74 Mb of *C. alburnus* sequences. *De novo* sequencing identified 31,279 protein-coding genes, with an average gene length of 8507.7 bp and average CDS length of 1115.23 bp. In addition, a high-density genetic linkage map consisting of 24 linkage groups was constructed based on 353,532 high-quality SNPs and 4,710 bin markers. A total of 28 QTLs corresponding to 11 genes, 26 QTLs corresponding to 11 genes and 12 QTLs corresponding to 5 genes were identified for sex, intermuscular spine length and body weight traits, respectively. These genetic markers or candidate genes associated with growth traits provide a basis for marker-assisted selection in *C. alburnus.*

## Data Availability

The datasets presented in this study can be found in online repositories. The names of the repository/repositories and accession number(s) can be found in the article/Supplementary Material.

## References

[B1] Aris-Brosou S. Yang Z. (2002). Effects of models of rate evolution on estimation of divergence dates with special reference to the metazoan 18S ribosomal RNA phylogeny. Syst. Biol. 51 (5), 703–714. 10.1080/10635150290102375 12396585

[B2] Blair J. E. Hedges S. B. (2005). Molecular phylogeny and divergence times of deuterostome animals. Mol. Biol. Evol. 22 (11), 2275–2284. 10.1093/molbev/msi225 16049193

[B3] Botstein D. White R. L. Skolnick M. Davis R. W. (1980). Construction of a genetic linkage map in man using restriction fragment length polymorphisms. Am. J. Hum. Genet. 32 (3), 314–331. 6247908 PMC1686077

[B4] Cádiz M. I. López M. E. Díaz-Domínguez D. Cáceres G. Yoshida G. M. Gomez-Uchida D. (2020). Whole genome re-sequencing reveals recent signatures of selection in three strains of farmed Nile tilapia (*Oreochromis niloticus*). Sci. Rep. 10 (1), 11514. 10.1038/s41598-020-68064-5 32661317 PMC7359307

[B5] Calanche J. Tomas A. Martinez S. Jover M. Alonso V. Roncalés P. (2019). Relation of quality and sensory perception with changes in free amino acids of thawed seabream (*Sparus aurata*). Food Res. Int. 119, 126–134. 10.1016/j.foodres.2019.01.050 30884640

[B6] Chen M. Zou M. Yang L. He S. (2012). Basal jawed vertebrate phylogenomics using transcriptomic data from Solexa sequencing. PLoS One 7 (4), e36256. 10.1371/journal.pone.0036256 22558409 PMC3338709

[B7] Conesa A. Götz S. García-Gómez J. M. Terol J. Talón M. Robles M. (2005). Blast2GO: A universal tool for annotation, visualization and analysis in functional genomics research. Bioinformatics 21, 3674–3676. 10.1093/bioinformatics/bti610 16081474

[B8] Delsuc F. Philippe H. Tsagkogeorga G. Simion P. Tilak M. K. Turon X. (2018). A phylogenomic framework and timescale for comparative studies of tunicates. BMC Biol. 16(1), 39. 10.1186/s12915-018-0499-2 29653534 PMC5899321

[B9] dos Reis M. Thawornwattana Y. Angelis K. Telford M. J. Donoghue P. C. Yang Z. (2015). Uncertainty in the timing of origin of animals and the limits of precision in molecular timescales. Curr. Biol. 25(22), 2939–2950. 10.1016/j.cub.2015.09.066 26603774 PMC4651906

[B10] Dudchenko O. Batra S. S. Omer A. D. Nyquist S. K. Hoeger M. Durand N. C. (2017). *De novo* assembly of the *Aedes aegypti* genome using Hi-C yields chromosome-length scaffolds. Science 356 (6333), 92–95. 10.1126/science.aal3327 28336562 PMC5635820

[B11] Dudchenko O. Shamim M. S. Batra S. S. Durand N. C. Musial N. T. Mostofa R. (2018). “The Juicebox Assembly Tools module facilitates *de novo* assembly of mammalian genomes with chromosome-length scaffolds for under $1000,”. bioRxiv.

[B12] Durand N. C. Robinson J. T. Shamim M. S. Machol I. Mesirov J. P. Lander E. S. (2016). Juicebox provides a visualization system for Hi-C contact maps with unlimited zoom. Syst 3 (1), 99–101. 10.1016/j.cels.2015.07.012 PMC5596920 27467250

[B13] Elinav E. Strowig T. Henao-Mejia J. Flavell R. A. (2011). Regulation of the antimicrobial response by NLR proteins. Immunity 34 (5), 665–679. 10.1016/j.immuni.2011.05.007 21616436

[B14] Feng X. Yu X. Fu B. Wang X. Liu H. Pang M. (2018). A high-resolution genetic linkage map and QTL fine mapping for growth-related traits and sex in the Yangtze River common carp (*Cyprinus carpio* haematopterus). BMC Genomics 19 (1), 230. 10.1186/s12864-018-4613-1 29609551 PMC5879560

[B15] Gold D. A. Runnegar B. Gehling J. G. Jacobs D. K. (2015). Ancestral state reconstruction of ontogeny supports a bilaterian affinity for Dickinsonia. Evol. Dev. 17 (6), 315–324. 10.1111/ede.12168 26492825

[B16] Kajitani R. Toshimoto K. Noguchi H. Toyoda A. Ogura Y. Okuno M. (2014). Effiient *de novo* assembly of highly heterozygous genomes from wholegenome shotgun short reads. Genome Res. 24 (8), 1384–1395. 10.1101/gr.170720.113 24755901 PMC4120091

[B17] Li H. Durbin R. (2010). Fast and accurate long-read alignment with Burrows-Wheeler transform. Bioinformatics 26 (5), 589–595. 10.1093/bioinformatics/btp698 20080505 PMC2828108

[B18] Li L. Stoeckert C. J. Roos D. S. (2003). OrthoMCL: Identification of ortholog groups for eukaryotic genomes. Genome Res. 13 (9), 2178–2189. 10.1101/gr.1224503 12952885 PMC403725

[B19] Li S. Chen X. Hao G. Geng X. Zhan W. Sun J. (2016). Identification and characterization of a novel NOD-Like receptor family CARD domain containing 3 gene in response to extracellular ATP stimulation and its role in regulating LPS-induced innate immune response in Japanese flounder (*Paralichthys olivaceus*) head kidney macrophages. Fish. Shellfish. Immun. 50, 79–90. 10.1016/j.fsi.2016.01.029 26820104

[B20] Li Y. Truc T. Wang W. (2010). Development of polymorphic microsatellite markers in topmouth culter (*Culter alburnus*). Conserv. Genet. Resour. 2 (1), 43–46. 10.1007/s12686-009-9157-x

[B21] Lich J. D. Williams K. L. Moore C. B. Arthur J. C. Davis B. K. Taxman D. J. (2007). Monarch-1 suppresses non-canonical NF-kappaB activation and p52-dependent chemokine expression in monocytes. J. Immunol. 178 (3), 1256–1260. 10.4049/jimmunol.178.3.1256 17237370

[B22] Liu B. Shi Y. Yuan J. Hu X. Zhang H. Li N. (2013). “Estimation of genomic characteristics by analyzing k-mer frequency in de novo genome projects,”. arXiv preprint arXiv, 13082012.

[B23] Liu S. L. Gu Z. M. Jia Y. Y. Zhao J. L. Jiang W. P. Li Q. (2014). Isolation and characterization of 32 microsatellite loci for topmouth culter (*Culter alburnus* Basilewsky). Genet. Mol. Res. 13 (3), 7480–7483. 10.4238/2014.September.12.14 25222247

[B24] Liu X. H. Stentiford G. D. Voronin V. N. Sato H. Li A. H. Zhang J. Y. (2019). *Pseudokabatana alburnus* n. gen. n. sp., (Microsporidia) from the liver of topmouth culter *Culter alburnus* (Actinopterygii, Cyprinidae) from China. Parasitol. Res. 118 (6), 1689–1699. 10.1007/s00436-019-06303-z 30976967

[B25] Mackay T. F. Stone E. A. Ayroles J. F. (2009). The genetics of quantitative traits: Challenges and prospects. Nat. Rev. Genet. 10 (8), 565–577. 10.1038/nrg2612 19584810

[B26] Meng L. Li H. Zhang L. Wang J. (2015). QTL IciMapping: Integrated software for genetic linkage map construction and quantitative trait locus mapping in biparental populations. Crop J. 3 (3), 269–283. 10.1016/j.cj.2015.01.001

[B27] Otsuka J. Sugaya N. (2003). Advanced formulation of base pair changes in the stem regions of ribosomal RNAs: Its application to mitochondrial rRNAs for resolving the phylogeny of animals. J. Theor. Biol. 222 (4), 447–460. 10.1016/s0022-5193(03)00057-2 12781743

[B28] Pathare P. B. Roskilly A. P. (2016). Quality and energy evaluation in meat cooking. Food Eng. Rev. 8 (4), 435–447. 10.1007/s12393-016-9143-5

[B29] Qi P. Guo B. Xie C. Wu C. Lu S. Duan Y. (2013). Assessing the genetic diversity and population structure of *Culter alburnus* in China based on mitochondrial 16S rRNA and COI gene sequences. Biochem. Syst. Ecol. 50, 390–396. 10.1016/j.bse.2013.04.010

[B30] Qi P. Qin J. Xie C. (2015). Determination of genetic diversity of wild and cultured topmouth culter (*Culter alburnus*) inhabiting China using mitochondrial DNA and microsatellites. Biochem. Syst. Ecol. 61, 232–239. 10.1016/j.bse.2015.06.023

[B31] Rabosky D. L. Chang J. Title P. O. Cowman P. F. Sallan L. Friedman M. (2018). An inverse latitudinal gradient in speciation rate for marine fishes. Nature 559 (7714), 392–395. 10.1038/s41586-018-0273-1 29973726

[B32] Rabosky D. L. Santini F. Eastman J. Smith S. A. Sidlauskas B. Chang J. (2013). Rates of speciation and morphological evolution are correlated across the largest vertebrate radiation. Nat. Commun. 4, 1958. 10.1038/ncomms2958 23739623

[B33] Ren L. Li W. Qin Q. Dai H. Han F. Xiao J. (2019). The subgenomes show asymmetric expression of alleles in hybrid lineages of *Megalobrama amblycephala* × *Culter alburnus* . Genome Res. 29 (11), 1805–1815. 10.1101/gr.249805.119 31649058 PMC6836732

[B34] Ren L. Tan X. J. Xiong Y. F. Xu K. Zhou Y. Zhong H. (2014). Transcriptome analysis reveals positive selection on the divergent between topmouth culter and zebrafish. Gene 552 (2), 265–271. 10.1016/j.gene.2014.09.053 25267534

[B35] Rexroad C. E. Palti Y. Gahr S. A. Vallejo R. L. (2008). A second generation genetic map for rainbow trout (*Oncorhynchus mykiss*). BMC Genet. 9 (1), 74. 10.1186/1471-2156-9-74 19019240 PMC2605456

[B36] Roelants K. Fry B. G. Norman J. A. Clynen E. Schoofs L. Bossuyt F. (2010). Identical skin toxins by convergent molecular adaptation in frogs. Curr. Biol. 20 (2), 125–130. 10.1016/j.cub.2009.11.015 20045326

[B37] Servant N. Varoquaux N. Lajoie B. R. Viara E. Chen C. Vert J. (2015). HiC-pro: An optimized and flexible pipeline for Hi-C data processing. Genome Biol. 16 (1), 259. 10.1186/s13059-015-0831-x 26619908 PMC4665391

[B38] Shan T. Yuan J. Su L. Li J. Leng X. Zhang Y. (2020). First genome of the Brown alga *Undaria pinnatifida*: Chromosome-level assembly using PacBio and Hi-C technologies. Front. Genet. 11, 140. 10.3389/fgene.2020.00140 32184805 PMC7058681

[B39] Simão F. A. Waterhouse R. M. Ioannidis P. Kriventseva E. V. Zdobnov E. M. (2015). BUSCO: Assessing genome assembly and annotation completeness with single-copy orthologs. Bioinformatics 31, 3210–3212. 10.1093/bioinformatics/btv351 26059717

[B40] Stam P. (1993). Construction of integrated genetic linkage maps by means of a new computer package: JoinMap. Plant J. 3 (5), 739–744. 10.1111/j.1365-313X.1993.00739.x

[B41] Stamatakis A. (2014). RAxML version 8: A tool for phylogenetic analysis and post-analysis of large phylogenies. Bioinformatics 30 (9), 1312–1313. 10.1093/bioinformatics/btu033 24451623 PMC3998144

[B42] Sun Y. Li Q. Wang G. Zhu D. Chen J. Li P. (2015). Development of transcript-associated microsatellite markers in *Ancherythoculter nigrocauda* and cross-amplification in *Culter alburnus* . Genet. Mol. Res. 14 (4), 14286–14290. 10.4238/2015.november.13.12 26600486

[B43] Tao W. Zou M. Wang X. Gan X. Mayden R. L. He S. (2010). Phylogenomic analysis resolves the formerly intractable adaptive diversification of the endemic clade of east Asian Cyprinidae (Cypriniformes). PLoS One 5 (10), e13508. 10.1371/journal.pone.0013508 20976012 PMC2958143

[B44] Uchino T. Hosoda E. Nakamura Y. Yasuike M. Mekuchi M. Sekino M. (2018). Genotyping-by-sequencing for construction of a new genetic linkage map and QTL analysis of growth-related traits in Pacific bluefin tuna. Aquac. Res. 49 (3), 1293–1301. 10.1111/are.13584

[B45] Walker B. J. Abeel T. Shea T. Priest M. Abouelliel A. Sakthikumar S. (2014). Pilon: An integrated tool for comprehensive microbial variant detection and genome assembly improvement. PLoS One 9 (11), e112963. 10.1371/journal.pone.0112963 25409509 PMC4237348

[B46] Wang C. M. Lo L. C. Zhu Z. Y. Yue G. H. (2006). A genome scan for quantitative trait loci affecting growth-related traits in an F_1_ family of Asian seabass (*Lates calcarifer*). BMC Genomics 7, 274. 10.1186/1471-2164-7-274 17064422 PMC1634999

[B47] Wang W. Chen L. Yang P. Hou L. He C. Gu Z. (2007). Assessing genetic diversity of populations of topmouth culter (*Culter alburnus*) in China using AFLP markers. Biochem. Syst. Ecol. 35 (10), 662–669. 10.1016/j.bse.2007.04.008

[B48] Wang X. Gan X. Li J. Mayden R. L. He S. (2012). Cyprinid phylogeny based on bayesian and maximum likelihood analyses of partitioned data: Implications for Cyprinidae systematics. Sci. China Life Sci. 55 (9), 761–773. 10.1007/s11427-012-4366-z 23015124

[B49] Xiao J. Kang X. Xie L. Qin Q. He Z. Hu F. (2014). The fertility of the hybrid lineage derived from female *Megalobrama amblycephala* × male *Culter alburnus* . Anim. Reprod. Sci. 151 (1-2), 61–70. 10.1016/j.anireprosci.2014.09.012 25287724

[B50] Yang Z. (1997). Paml: A program package for phylogenetic analysis by maximum likelihood. Comput. Appl. Biosci. 13, 555–556. 10.1093/bioinformatics/13.5.555 9367129

[B51] Ye C. Hill C. M. Wu S. Ruan J. Ma Z. S. (2016). DBG2OLC: Efficient assembly of large genomes using long erroneous reads of the third generation sequencing technologies. Sci. Rep. 6, 31900. 10.1038/srep31900 27573208 PMC5004134

[B52] Zhang L. Hu J. Han X. Li J. Gao Y. Richards C. M. (2019). A high-quality apple genome assembly reveals the association of a retrotransposon and red fruit colour. Nat. Commun. 10 (1), 1494. 10.1038/s41467-019-09518-x 30940818 PMC6445120

