# Supplementary material for: Chromosome-scale assembly and quantitative trait locus mapping for major economic traits of the Culter alburnus genome using Illumina and PacBio sequencing with Hi-C mapping information
Source: Front Genet. 2023 May 25;14:1072506. doi: 10.3389/fgene.2023.1072506 (PMC10248148; doi:10.3389/fgene.2023.1072506)
Supplement: Supplementary file 2 [file Table1.docx]

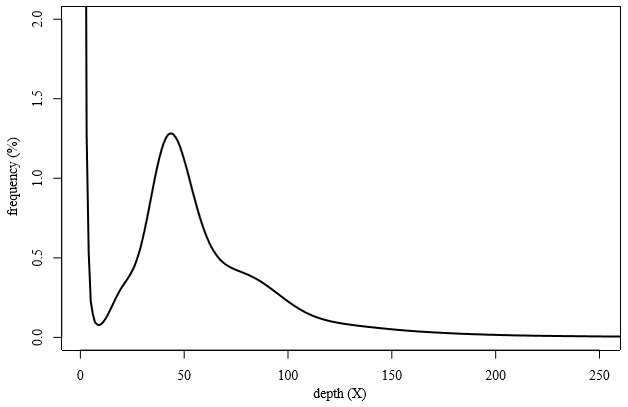


**Figure S1 The 17-mer count distribution for the genome size estimation. The x-axis in the figure is depth, and the y-axis is the proportion of K-mer types at each depth to all K-mer types.**


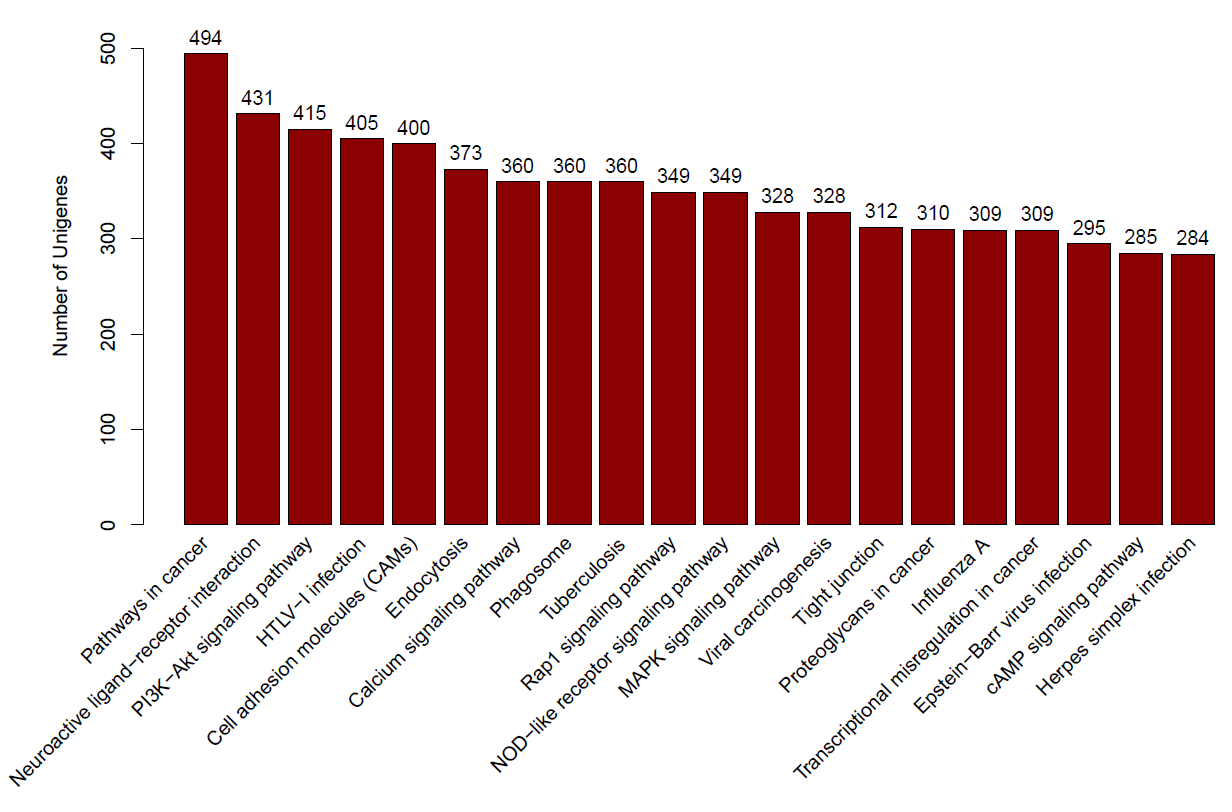


**Figure S2. The top 20 enriched KEGG pathways related with unigenes.**


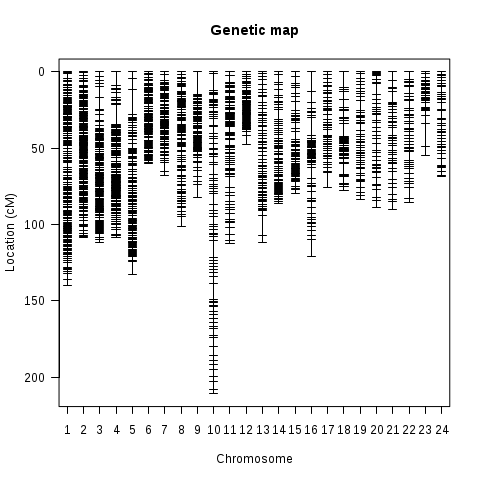


**Figure S3. Genetic linkage map of the *C. alburnus* constructed based on SNPs in “all” group**.


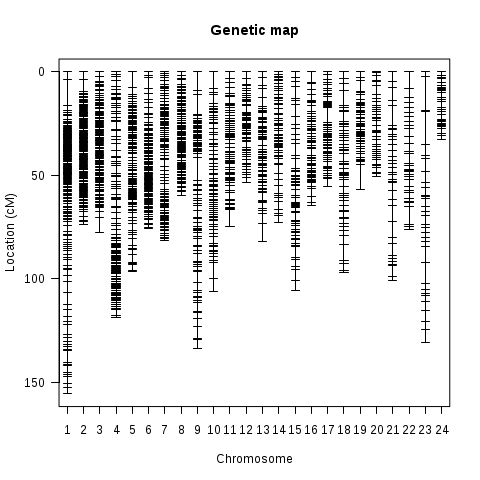


**Figure S4. Genetic linkage map of the *C. alburnus* constructed based on SNPs in “female” group.**


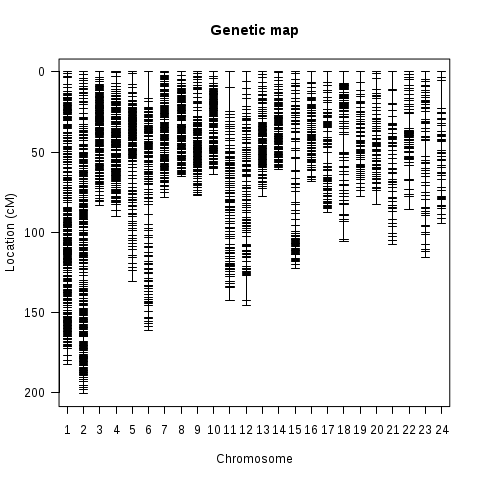


**Figure S5. Genetic linkage map of the *C. alburnus* constructed based on SNPs in “male” group (C).**
